# Relationally Charged: How and When Workplace Friendship Facilitates Employee Interpersonal Citizenship

**DOI:** 10.3389/fpsyg.2020.00190

**Published:** 2020-02-19

**Authors:** Jincen Xiao, Jih-Yu Mao, Jing Quan, Tao Qing

**Affiliations:** School of Business Administration, Southwestern University of Finance and Economics, Chengdu, China

**Keywords:** workplace friendship, relational energy, interpersonal citizenship, relational-interdependent self-construal, conservation of resources theory

## Abstract

Having friends in the workplace not only provides an employee joy and meaning, but also facilitates one’s positive behavior. In this study, we argue that workplace friendship has positive influence on an employee’s interpersonal citizenship behavior. Drawing upon conservation of resources theory, the present study explores how and when workplace friendship fosters interpersonal citizenship. Using a time-lagged, multisource data of 620 employees from 83 workgroups, we found that workplace friendship increases an employee’s relational energy, which subsequently, leads to greater interpersonal citizenship. Moreover, we discovered relational-interdependent self-construal as an important moderating influence that affects the saliency of this relationship. Specifically, for employee with a relational-interdependent self-construal, workplace friendship has a stronger positive influence on one’s relational energy and hence interpersonal citizenship. Contributions to theory and practice are also discussed.

## Introduction

Does having friends at work matter? How and why can friends affect a focal employee’s behavioral outcome? According to a study conducted by Gallup, people who have a best friend at work are not only to be happier and healthier, they are also seven times more likely to be engaged in their jobs as compared to those who say otherwise (Rath, 2006). In reality, friendships are private interpersonal networks that widely exist in an organizational setting (Ingram and Zou, 2008). Workplace friendship describes the quality of interpersonal relationship between individuals in the workplace where the relationship is characterized by mutual trust, commitment, reciprocal liking, and shared interests or values, which are driven by communal norms and socioemotional goals (Berman et al., 2002; Pillemer and Rothbard, 2018). Organizational research has found workplace friendship to have positive influences on both the employees and organization such as enhanced job significance (Mao et al., 2012), team-member exchange (Tse et al., 2008), well-being (Craig and Kuykendall, 2019), and innovation (Lu et al., 2017).

Indeed, having friends at work not only can enrich and bring joy to an employee’s organizational life (Rawlins, 1992; Sias and Cahill, 1998), but also can enable one to achieve a more successful career (e.g., Winstead et al., 1995; Ingram and Zou, 2008; Mao et al., 2012; Liu et al., 2013; Methot et al., 2016; Lu et al., 2017). Existing studies have found that workplace friendship can facilitate an individual’s psychological and behavioral responses by enhancing one’s affective experiences, such as happiness, excitement, trustworthiness, joy, and sympathy (e.g., Liu et al., 2013; Methot et al., 2016; Lu et al., 2017). Since friendship often elicits many of these positive emotions simultaneously, solely focusing on one particular emotion may not provide sufficient knowledge to understand the full picture of the positive influences of workplace friendship (Wright, 1984; Sias and Cahill, 1998; Ingram and Zou, 2008). Rather, these positive emotions form a collective force and provide the energy necessary to propel an individual’s action (Quinn and Dutton, 2005; Fritz et al., 2011; Cole et al., 2012; Baker, 2019). Hence, in this study, we investigate the consequences of workplace friendship from an energy perspective, as the arousal and accumulation of affective experiences are important sources of individual energy. To explore this promising line of research, we adopt conservation of resources (COR) theory to develop our theoretical rationale because energy is seen as a valuable resource that facilitates an employee’s behavior at work (Hobfoll, 1989).

Resource is something that a person values and can help an individual to attain one’s goals (Halbesleben et al., 2014). One such goal related to workplace friendship that an individual is likely to strive for is to develop and maintain interpersonal relationships (Sias and Cahill, 1998). This is because in a work context, friendships are likely developed from interpersonal interactions that characterize mutual support, help, and consideration in order to establish trust, liking, and common interests (Berman et al., 2002; Ingram and Zou, 2008; Pillemer and Rothbard, 2018). Hence, we suggest that positive interactions with friends are likely to stimulate an employee’s interpersonal citizenship behavior, and the key underlying influence is one’s elevated energy.

As a heightened level of affective state, energy is seen as a renewable resource that connects workplace events and related outcomes (Quinn and Dutton, 2005; Cole et al., 2012; Quinn et al., 2012). For example, subordinates become energized when they receive a sense of calling and membership from their spiritual leaders, which in turn, make them more capable of performing their jobs (Yang et al., 2019). In this study, we are particularly interested in relational energy, which is defined as “a heightened level of psychological resourcefulness generated from interpersonal interactions that enhances one’s capacity to do work” (Owens et al., 2016, p. 37), as its focus on energy generated from interpersonal interactions is in accordance with the interpersonal nature of workplace friendship. In essence, relational energy captures energy derived from social interactions (Baker, 2019).

Furthermore, the extent to which employees are affected by interpersonal relationships are dependent on how they construe themselves in relation to others (Gore et al., 2006; Morry and Kito, 2009; Cristina-Corina, 2012). For individuals with a relational-interdependent self-construal (RISC), they tend to emphasize their connectedness with others and strengthen existing relationships (Cross et al., 2000; Cross and Morris, 2003). Therefore, they highly value and are attentive to and affected by friends in the workplace (Gore et al., 2006). Consequently, we expect that employees with a RISC are more likely to be energized by workplace friendships.

**FIGURE 1:**
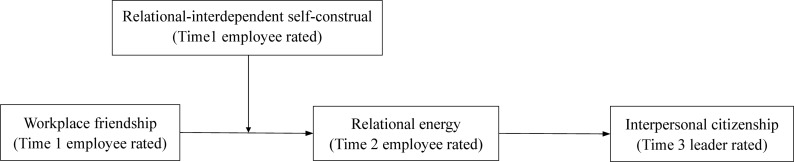
The proposed model of current research.

By testing these propositions in a time-lagged multisource study, our investigation of the impacts of workplace friendship through the lens of COR theory offers several contributions to the literature. First, instead of examining specific emotions in facilitating work-related outcomes of workplace friendship, our research differs from previous studies by focusing on the accumulation of positive emotions as a source of energy that motivates an employee’s interpersonal behavior (e.g., Winstead et al., 1995; Methot et al., 2016; Lu et al., 2017). In doing so, we approach from a different perspective and enhance understanding on the consequences of workplace friendship. Second, we identify relational energy as the key linking pin between workplace friendship and interpersonal citizenship. This finding sheds light on how workplace friendship influences interpersonal citizenship by suggesting that individuals can derive relational energy from workplace friends, which subsequently, drives them to help others. We also empirically answer Owens et al.’s (2016) call for exploring coworkers as a viable source of relational energy, as existing research has primarily associated relational energy with leadership behavior (e.g., Wang et al., 2018; Yang et al., 2019). Third, we incorporate RISC to understand how individual relational energy and thus demonstration of interpersonal citizenship as consequences of workplace friendship are affected by one’s interpersonal tendency. As such, we discover a contingency and integrate literatures on RISC and relational energy to better understand the consequences of workplace friendship. Altogether, the current study is at the forefront of exploring the impacts of workplace friendship through the lens of COR theory. Figure 1 presents our proposed research model.

## Theory and Hypotheses

### Workplace Friendship and Interpersonal Citizenship

Friendship is defined as “a relationship involving voluntary or unconstrained interaction in which the participants respond to one another personally, that is, as unique individuals rather than as packages of discrete attributes or mere role occupants” (Wright, 1984, p. 119). We suggest that workplace friendship is likely to encourage a focal employee to display interpersonal citizenship at work because interpersonal citizenship is also driven by voluntariness and socioemotional goal (Lam et al., 2016; Pillemer and Rothbard, 2018). Interpersonal citizenship occurs when employees assist others beyond their job requirements (Van Scotter and Motowidlo, 1996), and we expect that a focal employee would demonstrate interpersonal citizenship toward both friends and common work peers.

According to COR theory, social relationships are seen as a resource to the extent that they can provide valued resources (Hobfoll et al., 2018). As an overall indication of close interpersonal relationships in the workplace, workplace friendship can provide psychological and behavioral support (e.g., trustworthiness, information sharing) that are conducive to work (Sias and Cahill, 1998; Sias et al., 2012; Methot et al., 2016). In other words, interacting with friends at work can be emotionally fulfilling and can enrich employees’ psychological resource (Lilius, 2012; Lam et al., 2016; Craig and Kuykendall, 2019). Workplace friendship can motivate employees to show interpersonal citizenship because of the harmonious, supportive, and friendly environment that the individual is immersed in (Van Scotter and Motowidlo, 1996; Kim et al., 2013; Lam et al., 2016). Interpersonal citizenship at work includes cooperative, considerate, and helpful acts that assist coworkers’ performance (Van Scotter and Motowidlo, 1996), which is in accordance with the characteristics that workplace friendship resembles. Moreover, interpersonal citizenship is a positive interpersonal activity that enhances an individual’s positive affect (Koopman et al., 2016), facilitates social cohesiveness, and builds reciprocal ties with coworkers (Halbesleben and Wheeler, 2015). In particular, employees tend to have a desire to develop and progress with close friends, thus driving them to help their friends to finish tasks and achieve job goals (Sias and Cahill, 1998; Berman et al., 2002; Sias et al., 2012).

Furthermore, as Bowler and Brass (2006) noted that “it is not necessary for employees to help only those individuals that help them; they may pass on the help to others in a [pay it forward], pass it on manner” (p. 71). Similarly, interpersonal citizenship does not just happen between workplace friends, it can also be demonstrated to other common work peers. One important reason is that employees are inclined to enjoy and appreciate the benefits of workplace friendship, and thus display interpersonal citizenship to common work peers for the purpose of showing kindness and establishing bonding (Sias and Cahill, 1998; Bowler and Brass, 2006). For example, workplace friendship encourages mutual observational learning and advice seeking (Lee and Duffy, 2019). Therefore, we hypothesize the following:

Hypothesis 1: Workplace friendship is positively related to interpersonal citizenship.

### Relational Energy as a Linking Pin

Energy is a resource that can increase an employee’s capacity of motivation and action, thereby enabling one to perform and attain goals (Hobfoll, 1989; Quinn et al., 2012; Hobfoll et al., 2018). Energy can be derived from external stimulus, internal cognition, and relational interactions (Cole et al., 2012; Baker, 2019). Relational energy refers to the specific energy generated from interpersonal interactions, which can influence an employee’s work outcomes (Owens et al., 2016; Wang et al., 2018; Yang et al., 2019). For example, employees can receive relational energy when interacting with their humble leaders because the behaviors entailed in expressed humility represent social cues that can be interpreted as a form of non-material social giving (e.g., giving esteem and license), which can subsequently enhance their job performance (Wang et al., 2018). We argue that friends at work can arouse employees’ relational energy and then motivate them to display interpersonal citizenship.

Both of theoretical arguments and empirical evidences indicate that workplace friendship can foster relational energy. Friends, who provide trust, help, sense of meaning, and support at work, can be considered as a resource provider. Workplace friendship depicts an interactive schema of high interactional frequency and intense emotional injection (Jehn and Shah, 1997; Sias and Cahill, 1998; Ingram and Zou, 2008; Mao et al., 2012). Such relationship implies frequent face-to-face interactions wherein friends develop a mutual focus of attention and become entrained in each other’s bodily micro rhythms and emotions (Collins, 2004). In addition, social contagion between friends is particularly salient, providing a potential mechanism in which human energy can be transmitted through social interactions (Hatfield et al., 1994; Owens et al., 2016). For example, studies have shown that working alongside friends can engender happiness and positive affect (Sias and Cahill, 1998; Lu et al., 2017). Sense of meaning can also be derived from workplace friendship (Rawlins, 1992), and is closely related to energy (Fritz et al., 2011).

Moreover, we propose that an employee with high relational energy is more likely to demonstrate interpersonal citizenship behavior, as energy derived from interpersonal interactions is particularly important in maintaining interpersonal relationships (Owens et al., 2016; Baker, 2019). More broadly speaking, energized employees have a stronger willingness and capacity to show interpersonal citizenship. First, energized individuals are interested in building a broader interpersonal network (Fritz et al., 2011; Cullen-Lester et al., 2016). To that end, employees are likely to show their interpersonal citizenship, as it is a way to convey their kindness and friendliness. Second, relational energy represents a heightened level of psychological resourcefulness that captures the motivation, vitality, stamina, and vigor that is generated from interpersonal exchange (Owens et al., 2016). Individuals with relational energy have abundant resources that can afford them to engage in other behaviors such as helping and caring (Halbesleben et al., 2014; Trougakos et al., 2015). Therefore, we hypothesize the following:

Hypothesis 2: Relational energy mediates the positive relationship between workplace friendship and interpersonal citizenship.

### The Moderating Role of RISC

Self-construal studies suggest that RISC is an important personality trait that can greatly influence interpersonal preferences and processes (Cross et al., 2000; Cross and Morris, 2003; Morry and Kito, 2009; Baker and McNulty, 2013). Employees with a RISC usually prioritize goals involving developing and maintaining the connectivity of self-defining relationships (Heintzelman and Bacon, 2015). These employees are more likely to consider others in the decision-making process (Gore et al., 2006), which promote communal norms such as enacting appropriate behaviors and fostering interpersonal harmony (Baker and McNulty, 2013). Since workplace friendship affects one’s definition of the self, employees with different self-construals would interact with friends at work differently and thus are affected by friendship differently. As a result, the extent to which employees generate relational energy from workplace friendship is largely dependent on how they construe themselves in relation to others (Cristina-Corina, 2012).

Employees with a high RISC tend to place more value on connection and interdependence with others than those low in RISC (Gore et al., 2006). They are encouraged to promote and maintain interactions with others by engaging in behaviors such as accounting for the needs and wishes of others and empathizing others’ feelings (Cross et al., 2000; Cross and Morris, 2003). We propose that higher RISC can strengthen the relationship between workplace friendship and relational energy. First, as employees with a high RISC are more sensitive to others’ feelings and behaviors, they are more affected by interpersonal influences which allow them to establish a higher awareness of information exchange (Gore et al., 2006; Baker and McNulty, 2013). Second, social support from friends is especially valued by those with high RISC (Heintzelman and Bacon, 2015) because these individuals tend to experience more intense and emotional feelings from interpersonal interactions (Cross et al., 2000). Third, higher RISC is related to higher disclosure, which increases the frequency of interactions among friends (Morry and Kito, 2009). For these reasons, employees with a high RISC are more likely to be energized when interacting with friends.

In contrast, for employees with a low RISC, they do not view workplace friends as a vital factor in their self-construal (Cross and Morris, 2003). These individuals tend to derive self-meanings independent of social interactions, which means “Who am I” is likely to be answered with reference to internal traits that are stable across situations (Cross et al., 2011). As a result, their emotions and cognitions are less likely to be affected by others, which reduce the acquisition of relational energy from workplace friendship. Moreover, employees with a low RISC are more likely to judge workplace friendship as troublesome when behaviors required to fulfill instrumental goals conflict with socioemotional goals (Pillemer and Rothbard, 2018). Hence, the positive effect of workplace friendship on relational energy is reduced. Therefore, we hypothesize the following:

Hypothesis 3: RISC moderates the relationship between workplace friendship and relational energy, such that the relationship is stronger when RISC is high but weaker when it is low.

### A Moderated Mediation Model

The above hypotheses describe a picture of the relationship between workplace friendship and interpersonal citizenship behavior that suggests relational energy plays a mediating role and employees with a high RISC are likely to derive more relational energy from their friends in the workplace. Taken together, we propose a first-stage moderated mediation for the effect of workplace friendship on interpersonal citizenship.

Hypothesis 4: RISC moderates the indirect relationship between workplace friendship and interpersonal citizenship via relational energy, such that the indirect relationship is stronger when RISC is high rather than low.

## Materials and Methods

### Samples and Procedures

To test the theoretical model, we conducted a time-lagged (i.e., three time frames), multi-source (i.e., employees and their direct supervisors) survey study in a large public institution in southwestern China. Employees in this institution work in teams, and they have many opportunities to interact with each other in their daily work. The time-lagged, multi-source survey design was employed to minimize common method variance (Podsakoff et al., 2003). We asked the human resource department to randomly organize potential participating employees, where we solicited their on-site voluntary participation by explaining our research purpose, assuring the confidentiality of their responses, and providing office supplies and monetary incentives. Specifically, we gave each participant a card holder and a pen. In addition, we set up a lottery at the end of each survey questionnaire. Monetary rewards ranged from 1 to 100 Chinese Yuan. In total, 1047 employees from 150 workgroups expressed interests in participating our study.

In the time 1 questionnaire, 947 employees (response rate = 90.45%) from 132 workgroups returned their responses on workplace friendship, RISC, and demographic information. Two months later, we returned on-site and distributed the time 2 questionnaire, of which 856 employees (response rate = 81.76%) from 118 workgroups reported their relational energy. Another 2 months later, we revisited the company and distributed the time 3 questionnaire, of which 108 supervisors (response rate = 72.00%) answered their demographic information and assessed their subordinates’ interpersonal citizenship. After matching employees to their respective workgroup supervisors via a unique identification code, our final sample consisted of 620 employees and 83 supervisors.

Among the sampled employees, 56.77% were male, 68.55% had earned a bachelor degree or above, and 53.86% had an organizational tenure of at least 5 years. The average age was 34.40 (*SD* = 8.15). For the supervisor sample, 43.37% were male, 72.29% had earned a bachelor degree or above, and 83.13% had an organizational tenure of at least 5 years. The average age was 37.17 (*SD* = 4.73).

### Measures

All of the scales used to measure the main variables were originally developed in English. We followed Brislin’s (1980) translation and back-translation procedures to generate a Chinese version of measures. All of the main items were measured on a 5-point Likert scale (1 = strongly disagree to 5 = strongly agree). We calculated McDonald’s Omega coefficient to test the reliability of each scale (McDonald, 1999), which has been suggested to be a sensible index of internal consistency (Zinbarg et al., 2005).

#### Workplace Friendship

Workplace friendship was rated on the 12-item scale developed by Nielsen et al. (2000) in the time 1 questionnaire. An example item was “Being able to see my coworkers is one reason why I look forward to my job” (McDonald’s Omega = 0.93).

#### Relational-Interdependent Self-Construal

Relational-interdependent self-construal was assessed on the 11-item scale generated by Cross et al. (2000) in the time 1 questionnaire. A sample item was “My close relationships are an important reflection of who I am” (McDonald’s Omega = 0.98).

#### Relational Energy

Relational energy was measured on the 5-item scale developed by Owens et al. (2016) in the time 2 questionnaire. We replaced “this person” to “my friends at work.” Such operation can also be found in Wang et al. (2018) and Yang et al. (2019). An example item was “I feel invigorated when I interact with my friends at work” (McDonald’s Omega = 0.88).

#### Interpersonal Citizenship

Supervisors provided the ratings for employee interpersonal citizenship using the 7-item scale adopted by Van Scotter and Motowidlo (1996) in the time 3 questionnaire. A sample item was “This employee helps someone without being asked” (McDonald’s Omega = 0.92).

### Analytic Technique

Our data reflected a nested structure, with employees nested in workgroups led by a supervisor. Therefore, we used multilevel methods (i.e., random intercept models) to test the hypotheses at the employee-level of analysis (i.e., level 1), while taking possible group-level influence into account (i.e., level 2; Deng et al., 2018). Specifically, we used Mplus 7.4 (Muthén and Muthén, 2017) and employed the “cluster” and “type = two level random” syntaxes to perform the analyses. All independent variables were group-mean centered. To test for mediation effect, we adopted Selig and Preacher’s (2008) Monte Carlo method to derive coefficient estimates and confidence intervals (CIs) at 95% significance level with 20,000 sample repetitions. The Monte Carlo method has been suggested as a superior method than methods that rely on single point estimates (e.g., Sobel test; Preacher et al., 2010). To test for moderation effect, we evaluated the effect at “high” (one standard deviation above the mean) and “low” (one standard deviation below the mean) values of the moderator.

## Data Analysis and Results

### Confirmatory Factor Analysis

We conducted a series of confirmatory factor analyses to test the discriminant validity of our measurement model. As shown in Table 1, the fit indices of the hypothesized four-factor model are acceptable: chi-square (χ*^2^*) = 1522.82, degrees of freedom (*df*) = 550, *p* < 0.001; root mean square error of approximation (RMSEA) = 0.05; comparative fit index (CFI) = 0.93; Tucker–Lewis index (TLI) = 0.92; standardized root mean square residual (SRMR) = 0.05. The four-factor model is superior to any other alternative models. Hence, construct distinctiveness of the main variables is established.

**TABLE 1:** Comparison of measurement models.

**Models**	**χ^2^**	**df**	**χ^2^/df**	**△χ^2^ (df)^a^**	**RMSEA**	**CFI**	**TLI**	**SRMR**
Hypothesized 4-factor model (WF, RISC, RE, IC)	1522.82	550	2.77	—	0.05	0.93	0.92	0.05
Alternative 3-factor model (WF, RISC, RE + IC)	2365.79	553	4.29	842.97*** (3)	0.07	0.87	0.86	0.09
Alternative 3-factor model (WF, RISC + RE, IC)	2443.82	553	4.42	921.00*** (3)	0.07	0.86	0.85	0.10
Alternative 2-factor model (WF + RE + RISC, IC)	5371.45	555	9.68	3848.63*** (5)	0.12	0.64	0.62	0.19
Alternative 1-factor model (WF + RISC + RE + IC)	6871.53	556	12.36	5348.71*** (6)	0.14	0.53	0.50	0.21

*N* = 620 (employees), *N* = 83 (workgroups). WF, workplace friendship; RISC, relational-interdependent self-construal; RE, relational energy; IC, interpersonal citizenship; χ^2^, chi-square; df, degrees of freedom; RMSEA, root mean square error of approximation; CFI, comparative fit index; TLI, Tucker–Lewis index; SRMR, standardized root mean square residual. ^*a*^All models are compared with the hypothesized 4-factor model. ***p < 0.001.

### Hypothesis Tests

The means, standard deviations, inter-correlations, and internal consistencies of studied variables are presented in Table 2. Hypothesis 1 predicts a positive relationship between workplace friendship and interpersonal citizenship. As shown in Table 3, workplace friendship is positively related to interpersonal citizenship (*B* = 0.27, *SE* = 0.05, *p* < 0.001). Hence, Hypothesis 1 is supported.

**TABLE 2:** Means, standard deviations, inter-correlations, and internal consistencies of studied variables.

**Variables**	**Mean**	***SD***	**1**	**2**	**3**	**4**	**5**	**6**	**7**	**8**	**9**	**10**
(1) Employee gender	1.43	0.50	–									
(2) Employee age	34.40	8.15	0.11**	–								
(3) Employee education	2.88	0.73	–0.05	–0.06	–							
(4) Employee organizational tenure	2.91	1.41	0.06	0.66***	–0.07	–						
(5) Extraversion	3.86	0.77	0.01	–0.02	0.08*	–0.04	(0.92)					
(6) Positive affect	3.93	0.55	–0.00	–0.04	0.03	–0.06	0.43***	(0.92)				
(7) Workplace friendship	4.23	0.48	0.03	–0.05	–0.00	–0.01	0.09*	0.08	(0.93)			
(8) Relational energy	3.86	0.54	–0.02	–0.03	0.01	–0.06	0.18***	0.23***	0.39***	(0.88)		
(9) RISC	4.15	0.66	0.01	0.01	0.04	–0.03	0.04	0.04	0.20***	0.37***	(0.98)	
(10) Interpersonal citizenship	3.81	0.55	–0.04	0.03	0.07	0.03	0.21***	0.22***	0.23***	0.34***	0.12**	(0.92)

*N* = 620 (employees), *N* = 83 (workgroups). RISC, relational-interdependent self-construal; SD, standard deviation. McDonald’s Omegas are presented along the diagonal. Gender: 1 = male; 2 = female. Education: 1 = high school or below; 2 = junior college; 3 = bachelor; 4 = postgraduate. Organizational tenure: 1 = less than 1 year; 2 = 1–4 years; 3 = 5–8 years; 4 = 9–12 years; 5 = 13–16 years; 6 = 17 years or above. *p < 0.05, **p < 0.01, ***p < 0.001.

**TABLE 3:** Hierarchical linear modeling results.

**Variables**	**Main effect**	**Mediation effect**		**Moderation effect**
			
	**Interpersonal citizenship**	**Relational energy**	**Interpersonal citizenship**	**Relational energy**
Intercept	2.68*** (0.22)	3.86*** (0.02)	2.59*** (0.22)	3.84*** (0.02)
***Main predictors***				
Workplace friendship	0.27*** (0.05)	0.42*** (0.06)	0.12* (0.05)	0.36*** (0.06)
Relational energy			0.32*** (0.06)	
Relational-interdependent self-construal (RISC)				0.28*** (0.04)
***Interaction***				
Workplace friendship × RISC				0.30*** (0.06)

*N* = 620 (employees), *N* = 83 (workgroups). Values in parentheses are standard error estimates. *p < 0.05, ***p < 0.001.

Hypothesis 2 argues that relational energy mediates the relationship between workplace friendship and interpersonal citizenship. To test for mediation effect, we first obtained coefficient estimates by regressing relational energy on workplace friendship, and interpersonal citizenship on relational energy, respectively. Subsequently, we inserted these coefficient estimates into the Monte Carlo analysis (with 20,000 sample repetitions). The results from the Monte Carlo analysis indicate that relational energy mediates the positive relationship between workplace friendship and interpersonal citizenship (*indirect effect* = 0.13, *SE* = 0.03, *95% CI* = [0.08,0.19]). These findings provide support for Hypothesis 2.

**FIGURE 2:**
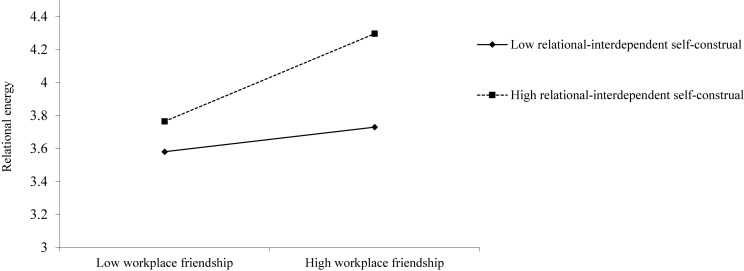
The interactive effect of workplace friendship and relational-interdependent self-construal on relational energy.

Hypothesis 3 predicts that RISC moderates the relationship between workplace friendship and relational energy, such that the relationship is stronger when RISC is high but weaker when it is low. As shown in Table 3, results reveal a significant interaction (*B* = 0.30, *SE* = 0.06, *p* < 0.001). Figure 2 illustrates the form of this interaction by plotting the simple slopes at “high” and “low” values of RISC. As shown in Table 4, first-stage effects indicate that the relationship between workplace friendship and relational energy is stronger in condition of high RISC (*B* = 0.56, *SE* = 0.07, *p* < 0.001), but weaker in condition of low RISC (*B* = 0.16, *SE* = 0.07, *p* < 0.05; *difference* = 0.40, *SE* = 0.09, *p* < 0.001). Hence, Hypothesis 3 is supported.

**TABLE 4:** First-stage, second-stage, and conditional indirect effect results.

**Outcome**	**Moderator**	**Stage**		**Effect**	
			
	**RISC**	**First (*P*_mx_)**	**Second (*P*_ym_)**	**Indirect (*P*_mx_ × *P*_ym_)**	**95% CI of indirect effect**
Interpersonal citizenship	Low (−1 *SD*)	0.16* (0.07)	0.32*** (0.06)	0.05* (0.03)	[0.004,0.10]
	High (+1 *SD*)	0.56*** (0.07)	0.32*** (0.06)	0.18*** (0.04)	[0.10,0.25]
	Difference	0.40*** (0.09)	–	0.13*** (0.03)	[0.06,0.19]

N = 620 (employees), N = 83 (workgroups). RISC, relational-interdependent self-construal; SD, standard deviation. *P*_mx_, path from workplace friendship to relational energy; *P*_ym_, path from relational energy to interpersonal citizenship. Values in parentheses are standard error estimates. Results from regression analyses were entered into the online utility developed by Selig and Preacher (2008). Monte Carlo resampling method (with 20,000 sample repetitions) was adopted to estimate confidence intervals (CIs) at 95% significance. *p < 0.05, ***p < 0.001.

**FIGURE 3:**
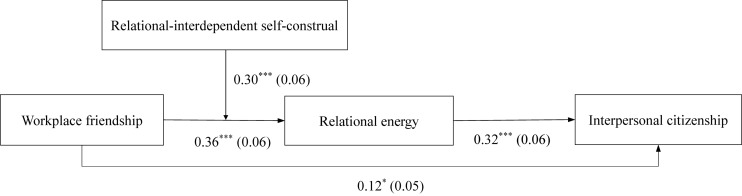
Path analysis results. ^∗^*p* < 0.05, ^∗∗∗^*p* < 0.001.

Hypothesis 4 theorizes that RISC moderates the indirect relationships between workplace friendship and interpersonal citizenship via relational energy, such that the indirect relationship is stronger when RISC is high rather than low. We investigated the indirect effects at “high” and “low” values of RISC. The results as well as the CIs generated from the Monte Carlo analysis (with 20,000 sample repetitions) are presented in Table 4. As indicated, the indirect effect of workplace friendship on interpersonal citizenship via relational energy is stronger when RISC is “high” (*indirect effect* = 0.18, *SE* = 0.04, *95% CI* = [0.10,0.25]), and weaker when it is “low” (*indirect effect* = 0.05, *SE* = 0.03, *95% CI* = [0.004,0.10]; *difference* = 0.13, *SE* = 0.03, *95% CI* = [0.06,0.19]). Altogether, these results provide support for Hypothesis 4. The path analysis results are presented in Figure 3.

### Supplementary Analysis

To ensure the robustness of our findings, we also included several control variables in our supplementary analysis. Previous research has pointed out that the effects of workplace friendship can be influenced by gender, as men tend to focus on shared activities while women emphasize shared emotions (Morrison, 2009). Consistent with existing research on workplace friendship, three other demographic variables were controlled, which are age, education, and organizational tenure (Chen et al., 2012; Methot et al., 2016). Considering that extraverts are more likely to develop and participate in interpersonal activities (Cullen-Lester et al., 2016; Methot et al., 2016), we controlled for employee extraversion using the Mini-IPIP scale (Donnellan et al., 2006; McDonald’s Omega = 0.92). In addition, people who have positive affectivity tend to experience more positive emotions (Vandenberghe et al., 2018), which may influence the level of relational energy. Thus, we also controlled for employee positive affect using Watson et al.’s (1988) Positive and Negative Affect Schedule (McDonald’s Omega = 0.92). In sum, we included control variables of gender, age, education, organizational tenure, extraversion, and positive affect in our supplementary analysis.

The results show a significant relationship between workplace friendship and interpersonal citizenship (*B* = 0.24, *SE* = 0.05, *p* < 0.001). In terms of the mediation effect, relational energy mediates the positive relationship between workplace friendship and interpersonal citizenship (*indirect effect* = 0.11, *SE* = 0.05, *95% CI* = [0.06,0.16]). Moreover, the moderating effect of RISC on the relationship between workplace friendship and relational energy is significant (*B* = 0.30, *SE* = 0.06, *p* < 0.001). Further, the indirect effect of workplace friendship on interpersonal citizenship via relational energy is stronger when RISC is “high” (*indirect effect* = 0.14, *SE* = 0.03, *95% CI* = [0.08,0.21]) than when RISC is “low” (*indirect effect* = 0.04, *SE* = 0.02, *95% CI* = [0.003,0.09]; *difference* = 0.11, *SE* = 0.03, *95% CI* = [0.05,0.16]). The results of the supplementary analysis show that the significance levels of all the hypothesized relationships remained the same when control variables were included (versus excluded) in the supplementary analysis, which suggests that our findings, to a large extent, are robust.

## Discussion

To better understand the influence of workplace friendship, scholars have increasingly called for nuanced perspectives that identify the process and outcomes of workplace friendship. In this research, we take a major step forward in this regard. That is, we adopt COR theory to explore how workplace friendship stimulates relational energy which fosters interpersonal citizenship, and the condition that affects the saliency of this relationship. Using data from 620 employees nested in 83 workgroups, we demonstrated that relational energy mediates the relationship between workplace friendship and interpersonal citizenship. Moreover, we found that this relationship is more salient for employees with a high RISC.

### Theoretical Implications

Our findings extend prior literature in multiple ways. First, in order to explore workplace friendship’s influence on interpersonal citizenship, we adopt COR theory to support our rationale. Recently, the COR perspective has been argued as a promising theoretical framework for studying the consequences of workplace friendship (Methot et al., 2016; Hood et al., 2017). The present study enhances understanding in this regard. Specifically, unlike previous studies that focused on an individual’s particular affective experience derived from interacting with one’s workplace friends, we treat affective experiences collectively as a source of energy that facilitates an individual’s interpersonal behavior. In doing so, we are able to find supporting evidence for elevated interpersonal citizenship behavior as a positive consequence of workplace friendship. In addition, previous research has often adopted a social exchange perspective to explain why positive interpersonal relationship can promote interpersonal citizenship (Farmer et al., 2015). However, this approach is more suitable in explaining interpersonal citizenship behavior directed back at the target of exchange, whereas it is less adequate in explaining interpersonal citizenship behavior demonstrated toward others (Cropanzano et al., 2017). The COR theory, on the other hand, provides a different perspective, as it suggests that individuals’ actions are affected by the acquisition of valued resource.

Second, we empirically test the COR framework by proposing and examining the mediating role of elevated resource in explaining the effect of workplace friendship on interpersonal citizenship. Specifically, we theorize relational energy as an important resource likely to be enhanced in response to workplace friendship, which further stimulates employees’ interpersonal citizenship behavior. Relatedly, existing literature has mainly linked relational energy to leader-subordinate interactions (Owens et al., 2016; Wang et al., 2018; Yang et al., 2019), because leaders, who are perceived as representatives of the organization, can energize their subordinates. We argue that friends in the workplace can also be a source that generates relational energy for a focal employee, as friends tend to interact with each other more and thus result in greater contagion of emotions. In doing so, we empirically answer Owens et al.’s (2016) call for investigating whether employee can receive relational energy from other interpersonal relationships besides one of leader-subordinate.

Third, our study highlights RISC as the boundary condition in the process of generation of relational energy and demonstration of interpersonal citizenship as consequences of workplace friendship. Support for the moderation effect provides novel and interesting insights for understanding how an interpersonal tendency can strengthen and weaken the consequences of workplace friendship. We found that when employees’ self-construal is more affected by social interactions, they are more likely to garner relational energy from their workplace friends, which subsequently, facilitates their interpersonal behavior. Other recent research has also begun to explore the boundary conditions of the influences of workplace friendship (e.g., Methot et al., 2016; Pillemer and Rothbard, 2018), suggesting that adopting an interactionist perspective is important and necessary to understand the influences of friendship in a workplace setting.

### Limitations and Future Research

The present study includes several limitations that highlight important avenues for future research. First, we encourage future scholars to investigate our theoretical model by adopting more rigorous methodological designs. Specifically, although we adopted a time-lagged, multi-source survey design, we are still not able to make strong causal inferences or rule out the possibility of reverse causality. For instance, employees who exhibit interpersonal citizenship may be more likely to generate relational energy or establish new workplace friendship. Thus, we encourage future research to adopt survey studies of a longitudinal design or experiment studies to address our model’s causality.

Second, our samples were drawn in a Chinese context. Research has suggested that the Chinese culture places great value on interpersonal relationship and harmony (Farh et al., 1998). Hence, workplace friendship may be more important and valued in a collectivist society, as opposed to in an individualistic society. In addition, it is not uncommon for employees of different cultural backgrounds to work together in a workplace environment (Khaleel et al., 2018). Since people of different cultural backgrounds can interpret workplace friendship differently and thus have different behavioral tendencies toward maintaining such friendship (Cross et al., 2000; Cross and Morris, 2003; Cristina-Corina, 2012), examining the consequences of workplace friendship in an integrated cultural context can also yield interesting and meaningful results. Therefore, we also encourage future studies to use different samples, preferably drawing samples from different cultural contexts, to examine the generalizability of our findings.

Third, recent research has begun to study workplace friendship using a social network approach (Methot et al., 2016; Hood et al., 2017; Tasselli and Kilduff, 2018). This approach highlights that an individual is “in the thick of things,” and that interpersonal relationships are more dynamic in nature (Methot et al., 2016). Furthermore, such approach has the advantage of moving analysis beyond the employee’s general perception of workplace friendship to a consideration of the structure and characteristics of the various relationships that the employee entertains in the workplace (Hayton et al., 2012). Specifically, the social network approach provides two network models (i.e., relational model and positional model) to explain the social influencing process. Relational model implies that social influence is operated through the mechanisms of cohesion and solidarity, where individuals are influenced by relational others. That is, individuals enjoy close social proximity with those whom they directly interact (Burkhardt, 1994). On the other hand, positional model emphasizes that individuals tend to pay attention to people whose positions are similar to those of theirs in the informal social structure (Burkhardt, 1994). Considering that individuals have direct interactions and enjoy close social proximity with friends in the workplace, we suggest future studies to adopt the relational model of social network to conceptualize and capture the influences of workplace friendship. In this way, workplace friends can be regarded as an important source of information, help, and support (Ho and Levesque, 2005).

Finally, we also urge future studies to adopt different theoretical lens to explore and enrich the consequences of workplace friendship. For instance, drawing upon optimal distinctiveness theory, high-quality interpersonal relationships can promote helping behavior through enhanced identification with exchange partners (Farmer et al., 2015). Self-determination theory may be another potential theoretical framework, as it can explain the relationship between workplace friendship and work-related outcomes from the perspective of basic psychological needs (Gagné and Deci, 2005). Furthermore, while we explored an individual difference as a boundary condition of the relationship between workplace friendship and relational energy, we suggest that contextual influences can also be critical contingencies worthy of investigation. For example, as a mechanistic unit structure is characterized by centralization of control and authority and extensive specialization and standardization of task (Aryee et al., 2008), it may reduce the frequency of interactions among friends, thereby weakening the relationship between workplace friendship and energy derived from interpersonal interactions.

### Managerial Implications

Workplace friendship is more prevalent nowadays because of the flatter organizational hierarchy which facilitates social interactions (Ingram and Zou, 2008). Our study provides important implications for managerial practice. First, our findings reveal that workplace friendship has constructive influence on employees’ interpersonal citizenship behavior, which suggests that developing and maintaining workplace friendship are important and conducive to employees’ interpersonal behavior. Hence, we suggest managers to foster work climates that facilitate the development of workplace friendship. They can organize more team-building activities and promote teamwork in order to increase employees’ opportunities to develop and enhance personal bonding. They can also facilitate interpersonal interactions among employees by, for example, assigning more team tasks and allocating more rewards to the achievement of these tasks.

Second, our findings show that relational energy is positively related to employee interpersonal citizenship. Previous studies also found that energy is a fuel that allows employees to self-motivate and engage in constructive work behavior (Quinn et al., 2012). Energized employees are able to develop more intimate relationships with their teammates, thereby contributing to team functioning through relevant behaviors such as information sharing (Sias et al., 2012; Cullen-Lester et al., 2016). Therefore, managers should be aware of and monitor employees’ energy levels (Schwartz and McCarthy, 2007). For example, managers can show more caring, trust, and encouragement to employees in their daily work. In doing so, their energy level can be enhanced, resulting in improved interpersonal citizenship.

Third, our findings reveal that workplace friendship exerts a stronger influence on employee interpersonal citizenship for those with a high RISC. This is possibly because that individuals with a high RISC tend to create a harmonious interpersonal climate when working with others. Thus, organizations are suggested to consider individual RISC as an essential criterion when selecting candidates for positions that require substantial teamwork. Moreover, organizations should allocate employees with an independent self-construal to positions that require fewer social interactions. Overall, organizations should emphasize the importance of interpersonal relationships during employee training and equip employees with necessary techniques to acquire and enhance relevant social skills.

## Conclusion

The present study investigates a moderated mediation model linking workplace friendship to employee interpersonal citizenship via relational energy. Moreover, we highlight the importance of RISC as a moderator affecting the consequences of workplace friendship. The moderated mediation model provides a comprehensive and clear understanding of the influences of friendship in the workplace. We hope that the theoretical and practical insights gained in this study will enrich understanding of workplace friendship and encourage researchers to further explore its consequences.

## Data Availability Statement

The dataset generated for this study is available upon request to the corresponding author.

## Ethics Statement

Ethical review and approval were not required for the study. Informed consent was obtained from all participants in the study.

## Author Contributions

JX designed and conducted the study and drafted the manuscript. J-YM and TQ edited the manuscript. J-YM and JQ conducted the data analysis. All authors discussed, finalized, and approved the manuscript for publication.

## Conflict of Interest

The authors declare that the research was conducted in the absence of any commercial or financial relationships that could be construed as a potential conflict of interest.
